# Nanopore Sequencing for Mycobacterium tuberculosis: a Critical Review of the Literature, New Developments, and Future Opportunities

**DOI:** 10.1128/JCM.00646-21

**Published:** 2022-01-19

**Authors:** Anzaan Dippenaar, Sander N. Goossens, Melanie Grobbelaar, Selien Oostvogels, Bart Cuypers, Kris Laukens, Conor J. Meehan, Robin M. Warren, Annelies van Rie

**Affiliations:** a Tuberculosis Omics Research Consortium, Family Medicine and Population Health, Institute of Global Health, Faculty of Medicine and Health Sciences, University of Antwerp, Antwerp, Belgium; b Unit of Mycobacteriology, Institute of Tropical Medicine, Antwerp, Belgium; c Department of Science and Innovation-National Research Foundation Centre for Excellence for Biomedical Tuberculosis Research, SAMRC Centre for Tuberculosis Research, Division of Molecular Biology and Human Genetics, Faculty of Medicine and Health Sciences, Stellenbosch University, Tygerberg, South Africa; d Department of Computer Science, University of Antwerp, Antwerp, Belgium; e Molecular Parasitology Group, Institute of Tropical Medicine, Antwerp, Belgium; f School of Chemistry and Bioscience, Faculty of Life Science, University of Bradford, Bradford, West Yorkshire, United Kingdom; Vanderbilt University Medical Center

**Keywords:** *Mycobacterium tuberculosis*, next-generation sequencing, Oxford Nanopore Technologies, nanopore sequencing, tuberculosis

## Abstract

The next-generation, short-read sequencing technologies that generate comprehensive, whole-genome data with single nucleotide resolution have already advanced tuberculosis diagnosis, treatment, surveillance, and source investigation. Their high costs, tedious and lengthy processes, and large equipment remain major hurdles for research use in high tuberculosis burden countries and implementation into routine care. The portable next-generation sequencing devices developed by Oxford Nanopore Technologies (ONT) are attractive alternatives due to their long-read sequence capability, compact low-cost hardware, and continued improvements in accuracy and throughput. A systematic review of the published literature demonstrated limited uptake of ONT sequencing in tuberculosis research and clinical care. Of the 12 eligible articles presenting ONT sequencing data on at least one Mycobacterium tuberculosis sample, four addressed software development for long-read ONT sequencing data with potential applications for M. tuberculosis. Only eight studies presented results of ONT sequencing of M. tuberculosis, of which five performed whole-genome and three did targeted sequencing. Based on these findings, we summarize the standard processes, reflect on the current limitations of ONT sequencing technology, and the research needed to overcome the main hurdles. The low capital cost, portable nature and continued improvement in the performance of ONT sequencing make it an attractive option for sequencing for research and clinical care, but limited data are available on its application in the tuberculosis field. Important research investment is needed to unleash the full potential of ONT sequencing for tuberculosis research and care.

## INTRODUCTION

Two decades after the genome sequence of the Mycobacterium tuberculosis strain H37Rv was published, sequencing technologies can generate comprehensive genomic data with unprecedented resolution, which makes them highly attractive for research, clinical care, and applications in tuberculosis (TB) control programs (1). While the implementation of M. tuberculosis sequencing has been facilitated by decreases in cost, technological advances, and improved bioinformatics to translate sequence data into biologically relevant information, the initial capital expenses of Illumina sequencing platforms, sequencing reagent costs, and the need for highly trained staff remain important hurdles for wide-spread implementation, especially in countries burdened by high levels of TB (2).

Long-read sequencing technologies are an enticing alternative to commonly used short-read sequencing platforms because it allows for the analysis of complex genomic loci and large repetitive elements, both distinct characteristics of the M. tuberculosis genome (1). Analyzing variation in these genomic regions could potentially provide a clearer understanding of genes involved in host-pathogen interactions and virulence. Moreover, nanopore sequencing, such as PacBio single-molecule real-time (SMRT) sequencing (Pacific Biosciences, Menlo Park, CA), can also identify methylation status (3), which is important because epigenetic modifications in M. tuberculosis have been associated with drug resistance, virulence, and regulation of gene expression profiles (4, 5). Nanopore sequencing platforms developed by Oxford Nanopore Technologies (ONT), are especially attractive due to their low cost and portable hardware. The ONT MinION (Oxford Nanopore Technologies, Oxford, United Kingdom) device can generate up to 30 gigabases (Gb) of long sequencing reads in 48 h in a decentralized laboratory (6). Recent reductions in error rates, updated flow cells, smaller amounts of required input DNA, and faster library preparation protocols have renewed the interest in its use in TB research and clinical applications (7–9).

We outlined the unique aspects of ONT sequencing and performed a critical narrative systematic review of the published literature on ONT sequencing of M. tuberculosis to reflect on recent developments and future opportunities.

## ONT SEQUENCING

**FIG 1:**
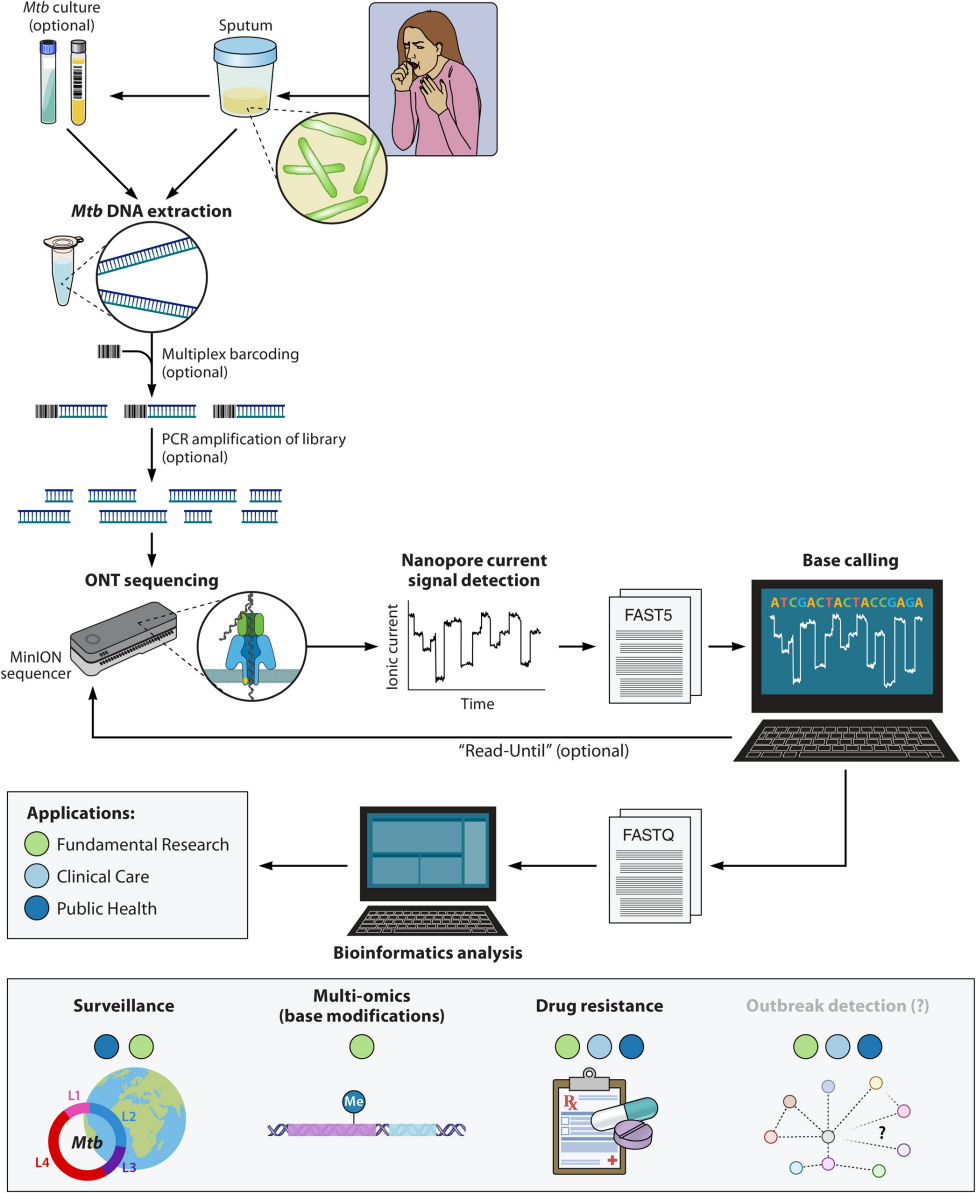
Overview of the Mycobacterium tuberculosis sequencing approach using Oxford Nanopore Technologies sequencing platform. After DNA extraction, usually from cultured M. tuberculosis but, in some cases, directly from clinical specimens, ONT library preparation is done, which may include barcoding and/or PCR amplification of the sequence library. The prepared library is loaded on the flow cell and inserted in the sequencer that is connected to a computer. During the ONT sequencing process, the current signal is detected, and the data are stored in the FAST5 format. If live base-calling is enabled, the optional and new Read-Until function can be used to selectively sequence nucleic acid molecules of interest. Base-called sequences are stored in the FASTQ format, which is analyzed using bioinformatics. M. tuberculosis ONT sequencing has applications in fundamental research, clinical care, and public health. Abbreviations: *Mtb*, Mycobacterium tuberculosis, ONT, Oxford Nanopore Technologies.

Nanopore sequencing is a unique, scalable technology that monitors changes in an electrical current as nucleic acids are passed through a nanopore protein. The resulting signal is decoded to provide the specific DNA or RNA sequence. The use of a nanopore for sequencing a single molecule of DNA or RNA negates the need for PCR amplification or chemical labeling of the sample for certain applications. The versatility of the platform and library preparation approaches allow for the sequencing of native nucleic acids, PCR libraries and amplified genomic targets. An overview of the general approach to the M. tuberculosis ONT sequencing process is shown in Fig. 1. In this section, we briefly describe the ONT flow cells, library preparation, base-calling, and bioinformatics analysis applicable to M. tuberculosis ONT sequencing.

### ONT flow cell chemistry.

ONT flow cells are designed to detect current signals from k-mers as the nucleic acid molecules move through the nanopore. The early R7 flow cell chemistry associated the presence of 6-mer nucleotides with the measured current signal, while this has been reduced to a 3-mer in R9 flow cells. Ignoring potential base modifications and assuming that only 4 different bases can be present corresponds to a reduction from 4096 (4^6^) possible k-mers to 64 (4^3^) possible k-mers during base-calling (10). In addition to pores that are occupied by shorter k-mers, dual reading of nucleotide sequences inside the pore was implemented in the latest R10 flow cells, implying that sequences are associated with a current signal at two different points in space and time inside the pore, resulting in an improved resolution and improved base-calling of homopolymeric regions.

### Library preparation.

A wide range of sequencing kits and library preparation approaches are available for ONT sequencing, each recommended for specific applications, and requiring various quantities of input material. For example, native DNA or RNA sequences can be detected in workflows without PCR amplification but require 400 to 1000 ng of input DNA (11, 12). Amplification-based approaches are recommended by ONT when input DNA is limited in quantity or quality, when control over the read length is required, and for targeted amplicon sequencing. Unless input DNA is limited, fragmentation of input DNA is not necessary, leading to a read length equal to the fragment length of the input DNA (9).

VolTRAX (Oxford Nanopore Technologies, Oxford, United Kingdom) is a portable automated sample preparation device that transforms biological samples into sequence-ready libraries and enables consistent library quality, even in the field or in the absence of elaborate laboratory infrastructure (9, 13, 14). VolTRAX is compatible with the hand-held MinION device and can multiplex up to 10 samples. ONT has also developed rapid field sequencing kits to overcome the challenges associated with cold-chain transport of reagents. Together, these developments increase the speed and simplicity of library preparation and reduce the need for specialized laboratory equipment and enhance the possibility of moving sequencing into the field and closer to point of care (15).

### Base-calling.

Oxford Nanopore Technologies’ sequencers monitor the changes in electrical current as nucleic acids pass through a nanopore protein. The resulting signals are stored in FAST5 files and are decoded during the ‘base-calling’ process, which translates the raw signals into nucleic acid sequences in FASTQ format (9). The electrical current signal reflects the presence of a k-length nucleotide sequence (k depending on the type of flow cell) passing through the nanopore. This makes base-calling of ONT sequencing data computationally more demanding, error prone, and complex than the a simple one-to-one conversion algorithm used by other sequencing technologies.

The first base-calling processes used hidden Markov models to estimate the likelihood that an observed signal corresponds to a particular k-mer sequence inside the nanopore (3). More recent base-callers use machine learning or hybrid models for inferring k-mers from raw signal data. Machine learning models are mainly trained on sequencing data derived from Escherichia coli, although several base-callers (such as Guppy (9) and Chiron (16)) allow users to train the algorithm *de novo*. Ideally, a base-caller for M. tuberculosis would be trained on M. tuberculosis sequencing data with a known ground truth (reference sequence) so that the base modifications found in M. tuberculosis (such as m6A and m5C [17]) and species-specific sequence patterns can be captured accurately.

### Bioinformatic analysis.

Bioinformatic analysis pipelines for M. tuberculosis ONT sequencing follow a similar approach to pipelines for short-read (e.g., Illumina) data, with quality control (read trimming), alignment to a reference genome, variant identification, and annotation to identify genomic variants for diagnosis of drug resistance or identification of transmission events (1). Some short-read pipelines (Mykrobe and TBProfiler) have already been updated to also analyze longer read sequence data (18, 19).

In contrast to most short-read pipelines that align sequence reads to the reference genome, ONT sequence analysis pipelines typically include the option to perform genome assembly *de novo* (e.g., Flye [20]). An interesting but costly approach is a hybrid assembly (21) in which the (more error prone) long reads are used to close gaps by linking contigs and resolving repeat regions and the (accurate) short reads are mapped to the assembled contigs to correct for sequencing errors. This approach has been used for M. tuberculosis (21) and is especially valuable for analysis of highly repetitive regions, such as *pe/ppe* regions, for detecting structural genome variation (e.g., inversion, insertions, or deletions), and for deciphering the genomes of novel M. tuberculosis lineages.

## ONT SEQUENCING FOR M. TUBERCULOSIS RESEARCH AND CLINICAL CARE

We performed a search in PubMed and Scopus on 20 Jan 2021 using the search terms “tuberculosis” or “Mycobacterium tuberculosis” and “nanopore” or “Oxford Nanopore Technologies” or “portable sequencing” without date or language restrictions. Papers were eligible for inclusion if M. tuberculosis ONT data were presented for at least one sample or if the development of bioinformatics tools for analysis of M. tuberculosis ONT data was described. We identified 58 articles of which 12 were eligible. Five articles focused on whole-genome sequencing (WGS) (8, 12, 21–23), three on targeted sequencing (Table 1) (11, 24, 25), and four on software development for long-read ONT sequencing data with applications for M. tuberculosis (Table 2) (16, 18, 19, 26).

**TABLE 1:** Publications using Oxford Nanopore Technologies sequencing data for Mycobacterium tuberculosis^*a*^

First author	Year	Ref	WGS or targeted	Type of strain, cultured isolate or specimen	N	Sample preparation details and library preparation kit	Device	Flow cell used	Base-calling	Bioinformatic analysis	Main study aim	Main study findings
Eckert	2016	22	WGS	Laboratory (*Mtb* H37Rv), culture isolate	1	Biotinylated RNA bait enrichment, SQK-MAP003 or SQK-MAP004	MinION	Not listed	Metrichor 2D	MinKNOW, poretools, BLASR, LAST	Evaluate an adapted DNA enrichment protocol for MinION sequencing	DNA enrichment resulted in partial *Mtb* genome coverage
				Clinical, cultured isolate	1							
Bainomugisa	2018	21	WGS	Clinical, cultured isolate	1	SQK-LSK108	MinION	R9.4	Albacore	MinKNOW, Nanopolish, Racon, Pilon, MUMmer, Canu, Circulator	Use NS plus short-read sequencing to assemble the XDR *Mtb* genome	Identification of known and novel genomic variants
Smith	2020	23	WGS	Clinical, cultured isolates	431	SQK-LSK109	MinION	R9.4	Guppy with FlipFlop Fast algorithm	QCAT, Minimap2, BWA mem, SAMTools, Kraken	Assess ONT sequencing for species identification, *in silico* spoligotyping, resistance prediction and phylogenetics	Performance and cost of ONT is comparable, to Illumina for genotyping and detection of resistance
Cervantes	2020	8	WGS	Laboratory (*Mtb* HN878), cultured isolate	1	Rapid Sequencing Kit	MinION	R9.4	Albacore	EPI2ME, What’s In My Pot, antimicrobial resistance mapping application	Evaluate ONT for WGS for drug resistance prediction from cultured and uncultured *Mtb*	no. of *Mtb* reads varied considerably and was very low for 2 when DNA was extracted directly from sputum
				Clinical, 4 cultured isolates, 2 specimens	6							
George	2020	12	WGS	Laboratory (M. bovis BCG), cultured isolate (used to spiked sputum)	1	SQK-LSK109	GridION	R9.4	Guppy	Porechop, Centrifuge, in-house CRuMPIT workflow, Minimap2, SAMTools, Pysam	Develop an undemanding, cost-effective method for sequencing *Mtb* directly from clinical specimens	Use of a low-cost thermo-protection buffer and a single flow cell per sample resulted in sufficient *Mtb* genome coverage
				Clinical, specimens	20							
Tafess	2020	24	Targeted	Clinical, cultured isolates	163	PCR amplification of 19 loci, SQK-LSK108	MinION	R9.4	Albacore	BacterioChek-TB, BWA	Develop targeted sequencing for Illumina MiSeq and ONT for prediction of resistance.	100% concordance between ONT and Illumina when low frequency variants are excluded
Chan	2020	25	Targeted	Clinical, specimens	12	PCR amplification of 10 loci, Ligation Sequencing 1D kit	MinION	R9	MinKNOW	Porechop, Minimap2, Nanopolish, Qualimap	Develop targeted sequencing workflows for Illumina MiSeq and ONT for prediction of resistance	95% concordance between ONT and Illumina for fixed variants
Cabibbe	2020	11	Targeted	Clinical, specimens	104	Deeplex Myc-TB PCR amplification, SQK-LSK108	MinION	R9.4	Albacore	Guppy, Porechop, Minimap2, SAMTools, VarScan2, NanoPack, AlignQC, Qualimap2	To evaluate the compatibility of Deeplex Myc-TB, with ONT MinION.	ONT MinION and Illumina MiniSeq results were fully concordant for drug resistance prediction.

a N refers to the number of sequenced samples. Twenty replicates of one sample. Abbreviations: WGS, whole-genome sequencing; BCG, Bacillus Calmette-Guérin; ONT, Oxford Nanopore Technologies; XDR, extensively drug resistant; *Mtb*, Mycobacterium tuberculosis.

**TABLE 2:** Published software for analysis of Mycobacterium tuberculosis Oxford Nanopore Technologies sequence data^*a*^

First author	Year	Reference	Name	Purpose	No. of samples
Hunt	2019	19	Mykrobe	Drug resistance prediction, species identification	5 *Mtb* clinical isolates
Phelan	2019	18	TBProfiler	Drug resistance prediction, *Mtb* lineage assignment	34 replicates of 3 *Mtb* clinical isolates
Teng	2018	16	Chiron	ONT sequencing base-caller	1 *Mtb* clinical isolate
Tang	2020	26	MIRUReader	*In silico* MIRU-VNTR from long-read *Mtb* sequencing data	15 *Mtb* clinical isolates

a All publications listed used M. tuberculosis WGS generated using an ONT MinION device. Abbreviations: *Mtb*, Mycobacterium tuberculosis; ONT, Oxford Nanopore Technologies; MIRU-VNTR, mycobacterial interspersed repetitive unit-variable number tandem repeat.

### Research applications.

The first ‘proof of principle’ study was published in 2016 and focused on the development of an enrichment protocol of preparation of M. tuberculosis DNA for ONT sequencing (22). Eckert et al. (22) mixed M. tuberculosis DNA (H37Rv and DNA from a clinical extensively drug-resistant strain) with human genomic DNA (at 10% and 90% M. tuberculosis DNA) and used biotinylated RNA baits synthesized based on M. tuberculosis H37Rv to capture long fragments of M. tuberculosis DNA. They reported that unenriched mixtures resulted in very low M. tuberculosis genome coverage while enrichment resulted in partial genome coverage. Areas with high coverage depth corresponded to open reading frames encoding transposases, which may reflect redundancy of the captured sequence.

In 2018, Bainomugisa et al. (21) used ONT sequencing to investigate a Beijing strain that had caused outbreaks of drug-resistant M. tuberculosis in Papua New Guinea. By combining a complete ONT-based genome assembly with Illumina sequencing for error correction, Bainomugisa et al. (21) identified all drug resistance-causing mutations and novel variation, including three previously undescribed genomic deletions (1315, 1355, 1356 bp, respectively), two insertions (390 and 4490 bp), multiple variants in repetitive *pe/ppe* gene regions, and compensatory mutations.

### Clinical applications.

The largest clinical study of 431 cultured isolates was published in 2020 (23). Smith et al. (23) aimed to validate ONT for species identification, *in silico* spoligotyping, detection of drug resistance, and phylogenetic analysis. Whole-genome sequencing on the ONT MinION showed drug resistance profiles comparable to those obtained by Illumina MiSeq (96% and 96,2% respective concordance with phenotypic drug susceptibility testing), and with equal or faster turnaround time and competitive per sample sequencing cost (±$63 for ONT versus $130 for Illumina MiSeq). Small insertions or deletions and heterozygous variants were more difficult to ascertain with high accuracy using ONT data.

A small study published in 2020 aimed to validate the ONT rapid sequencing kit for detection of drug resistance in M. tuberculosis (8). Cervantes et al. (8) observed that the number of reads aligned to the M. tuberculosis reference genome varied considerably (6,736 to 28,090) for purified DNA extracted from one laboratory and four clinical M. tuberculosis isolates. When DNA was extracted directly from two sputum specimens, the number of mapped M. tuberculosis reads was very low (16 and 53), and most of the reads produced corresponded to human DNA.

The first study of culture-free ONT sequencing of bronchioalveolar lavage and lymph node aspirate specimens was also published in 2020 (12). George et al. (12) achieved a mean genome coverage for M. tuberculosis clinical specimens ranging from 0.55× to 81×. A high (99.9%) consensus accuracy from ONT data was obtained when Nanopolish, a software package designed to analyze ONT data at the signal-level, was used. Unfortunately, multiplexing resulted in insufficient genome coverage because 5% to 47% of reads could not be reliably assigned to an input sample. Optimal ONT sequencing results were thus only achieved when one flow cell was dedicated to a single sample, rendering this approach prohibitively expensive for routine clinical settings.

Three studies that assessed ONT sequencing for targeted sequencing of drug resistance loci were all published in 2020 (11, 24, 25). Tafess et al. (24) and Chan et al. (25) used custom-made panels of 19 and 10 drug resistance-associated loci, respectively. Tafess et al. (24) showed 100% agreement in the detection of drug resistance between ONT and Illumina MiSeq when variants with an allele frequency below 40% reported by ONT sequencing were excluded. Chan et al. (25) showed 95% concordance for ONT-detected variants with an allele frequency of 100% as reported by MiSeq. The cost per sample for sequencing 19 loci with drug resistance as developed by Tafess et al. (24) was $72 on the ONT MinION and $68 on Illumina MiSeq, but the turnaround time was shorter using the MinION (15 h) compared to the Illumina MiSeq (38 h) (24). Chan et al. (25) reported similar per sample assay cost and sequencing costs on the ONT MinION device ($64) when sequencing 24 samples per flow cell (25). Cabibbe et al. (11) assessed the GenoScreen Deeplex Myc-TB assay and found full concordance in detecting drug-resistant variants between ONT MinION and Illumina MiniSeq when applying an allele frequency threshold of 80%. The assay and sequencing costs were comparable between the ONT MinION and Illumina MiniSeq, at approximately 100 euros per sample (11).

### Software development.

Two papers published in 2019 focused on the use of existing bioinformatic pipelines (Mykrobe [previously Mykrobe predictor] and TBProfiler) to process FASTQ files for M. tuberculosis ONT WGS data (18, 19). Mykrobe predictor was one of the first software packages for species identification and M. tuberculosis drug resistance prediction, but the use of Mykrobe predictor for ONT WGS analysis was not evaluated for M. tuberculosis (27). Hunt et al. (19) assessed the updated version of Mykrobe, which features an updated statistical model for ONT data, updated resistance library, and functionality to use a custom drug-resistant library on five M. tuberculosis isolates. Mykrobe detected the exact same drug resistance-causing mutations from both ONT and Illumina sequence data (19). TBProfiler, another bioinformatics tool developed to predict drug resistance and infer M. tuberculosis lineage and strain type from Illumina WGS data (28), was updated by Phelan et al. (18) and adapted to allow for the analysis of ONT data (18). TBProfiler analysis of 34 replicates of three multidrug-resistant M. tuberculosis isolates showed that one resistance-conferring variant (insertion in the *tlyA* gene) was missed during the analysis of the ONT MinION data compared to Illumina data (18).

Teng et al. (16), developed Chiron as an open-source base-calling algorithm. Chiron translates raw nanopore current signals directly into nucleotide sequences using deep learning neural networks (16). Chiron was trained on viral (Escherichia virus Lambda) and bacterial (Escherichia coli) sequencing reads and allows users to train the neural network within the software with their own specific genomes of interest (with distinct characteristics). When used for the base-calling of a single M. tuberculosis isolate sequenced with a MinION device, Chiron was shown to be more accurate than Albacore V1.1 and nearly as accurate as Albacore V2.0.1 (developed by ONT).

Finally, Tang et al. (26) performed a small validation study of the MIRUReader software to identify mycobacterial interspersed repetitive unit-variable number tandem repeat (MIRU-VNTR) typing profiles from ONT data. MIRUReader was able to predict the MIRU-VNTR profiles correctly from ONT MinION WGS data from 13 of the 15 M. tuberculosis strains that were assessed, and the profiles were identical to those obtained using the GenoScreen MIRU-VNTR Quadruplex kit.

## CRITICAL EVALUATION OF STRENGTHS AND LIMITATIONS OF M. TUBERCULOSIS ONT SEQUENCING

The key strengths of ONT’s sequencing platforms are that they are a low capital investment, have competitive per sample sequencing cost when multiplexing, the possibility of bias-free PCR library preparation, cold-chain-free sequencing reagents, fast turnaround time, use of long reads to resolve complex genomic loci, and the ability to investigate methylation status.

The main limitation of ONT sequencing remains the suboptimal accuracy. Sequencing accuracy can be expressed as single-read or as consensus accuracy. In 2015, the single-read accuracy (or percentage identity of a sequence compared to its reference sequence) of ONT data was only 60% (10). The low single-read accuracy was due to random read errors. Following changes in flow cell chemistry, improvements in base-calling software, development of post-sequencing correction tools, and 2D and 1D^2^ sequencing, the single-read accuracy has increased to >95% for the latest R10.3 flow cells (9). While this remains lower than the 99.9% accuracy of short-read Illumina sequencing (29), accuracy data for the latest R10 flow cells (post R10.3) has not yet been published. Consensus accuracy measures the identity of a consensus sequence constructed from multiple, overlapping reads originating from the same genomic location, and depends on systematic errors. For M. tuberculosis, the consensus accuracy for *de novo* genome assembly is estimated at 99.63% at 130× coverage and 99.92% at 238× coverage (16, 21). As a consensus accuracy of 99.63% would correspond to >15,000 errors in the 4.4 Mbp M. tuberculosis genome, the high false-positive rate of ONT sequencing remains a barrier for TB outbreak investigations. ONT is sensitive to errors in homopolymeric regions (10). When a stretch of identical k-mers passes the through the nanopore, a window of similar current signals is generated, which complicates the determination of the number of identical nucleotides that are present. Base-calling of homopolymeric regions larger than the k-mer length and recognized by the nanopore is therefore particularly challenging (30). This leads mostly to reduced ONT sequence accuracy for insertions and deletions.

The current accuracy levels achieved by ONT are likely sufficient to confidently detect drug resistance-conferring mutations (11, 18, 19, 21, 24), but may be suboptimal to detect heteroresistance or mixed infection, and to infer transmission events. For example, while Illumina WGS data can detect 1% to 3% heteroresistance at a depth of 400× and 100×, respectively (31), higher allele frequency thresholds (40% [24] and 80% [11]) for ONT data were required to achieve full concordance with Illumina sequencing for detection of drug resistance. Furthermore, detection of mixed infections in Illumina WGS data can be done accurately using QuantTB, which identifies mixed infections based on lineage-specific, single nucleotide, variant markers, but this tool has not been validated for ONT data.

The output and accuracy of ONT flow cells have improved over the past years. Nonspecific PCR-based library preparation of sputum spiked with M. bovis bacillus Calmette-Guerin (BCG)-purified DNA (5%, 10%, and 15%) that was sequenced on R9 and R9.4 ONT flow cells showed reduced coverage bias and higher data yields using the R9.4 ONT flow cells compared to the R9 ONT flow cells (7). In addition, the latest R10.3 ONT flow cells provide increased throughput, and have a longer signal detection area and, therefore, improved base-calling can be achieved. The release of the Flongle adapter in 2019 provides a low-output sequencing solution (2 Gb) at $90 per Flongle flow cell, which is the lowest setup cost of any sequencing platform currently available.

Finally, an important limitation to the application of ONT sequencing in M. tuberculosis research, clinical care, and public health lies in the limited experience with M. tuberculosis ONT sequencing to date because ONT data for only 764 M. tuberculosis strains has been published by January 20, 2021.

## FUTURE PROSPECTS

One of the most promising and recent ONT developments is the so-called “Read-Until” functionality of ONT sequencers. During Read-Until workflows, base-calling and rapid reference alignment are carried out in real time, while the DNA or RNA molecule is passing through the nanopore (9). This sequence information is then used to decide whether a particular molecule should be sequenced further or ejected preliminarily from the pore by reversing the voltage. Thus, Read-Until allows for the ability to direct more sequencing coverage toward targeted genomes or genomic regions that are of interest to the investigator. Read-Until currently reaches enrichments of 2.7× to 5.4× because ejecting the sequencing read comes with a small, but cumulative, risk of blocking the nanopore for the remainder of the sequencing run (32). Hence, an increase in selectivity will result in more pores being blocked during the sequencing process and a lower gigabyte output of the flow cell. Future improvements, such as onboard nucleases, that unblock the pores may resolve this problem. In regards to M. tuberculosis research and tuberculosis care, Read-Until could greatly advance the field of WGS directly from sputum or other clinical specimens because these samples typically have large amounts of human and microbial contaminant DNA compared to the low copy numbers of M. tuberculosis genomes (12). Additionally, Read-Until could be used to direct more sequencing coverage toward genes that confer drug resistance in M. tuberculosis.

Another area of TB research where ONT sequencing could play an important role is epigenetics. The low cost of the ONT hardware, development of open-source software to identify epigenetic base modifications, and long-read, PCR bias-free sequencing of native DNA and RNA make ONT sequencing an attractive alternative for future epigenetic and multiomics M. tuberculosis investigations (3), but these studies have not yet been performed. Studies using PacBio sequencing revealed that different methylation patterns may influence virulence, pathogenicity, and the development of (lineage-specific) drug resistance in M. tuberculosis (4). To date, only one study has used PacBio sequencing to combine genomic, transcriptomic, and methylation analysis of 22 M. tuberculosis isolates. Gomez-Gonzalez et al. (5) found a relationship between the DNA sequence, methylation, and RNA expression. Further research is needed to explore the multiomics potential of ONT sequencing research and to verify the functional consequences of the identified mechanisms of gene expression regulation.

## CONCLUSION

The low capital cost and portable nature of ONT hardware, the simplification and automation of sample and library preparation steps when using VolTRAX, and continuous improvements in sequencing accuracy suggest that ONT could have value in mycobacterium research laboratories, especially for detection of drug resistance. The development of the Read-Until function may accelerate researchers’ ability to sequence directly from sputum samples. Oxford Nanopore Technologies’ long-read sequencing may expand the research applications in M. tuberculosis sequencing beyond what is possible using short-read sequence analysis workflows by including epigenetics and investigations of the role of repetitive elements and complex regions of the M. tuberculosis genome. The lower per-base accuracy of ONT sequencing compared to that of Illumina technologies currently limits its use in the detection of transmission events and the study of heteroresistance or mixed infections. Experiences with its application in public health, clinical care, and M. tuberculosis research remain limited, and the lack of consensus in the bioinformatics analysis of M. tuberculosis ONT sequence data make its implementation in clinical care or public health laboratories premature.
